# Diet, Sports, and Psychological Stress as Modulators of Breast Cancer Risk: Focus on *OPRM1* Methylation

**DOI:** 10.3389/fnut.2021.747964

**Published:** 2021-12-08

**Authors:** Liangliang Li, Shuo Li, Shidong Qin, Yu Gao, Chao Wang, Jinghang Du, Nannan Zhang, Yanbo Chen, Zhen Han, Yue Yu, Fan Wang, Yashuang Zhao

**Affiliations:** ^1^Department of Epidemiology, School of Public Health, Harbin Medical University, Harbin, China; ^2^The Second Affiliated Hospital of Harbin Medical University, Harbin, China; ^3^The Third Affiliated Hospital of Harbin Medical University, Harbin, China

**Keywords:** breast cancer, risk, methylation, environmental factors, clinicopathological features

## Abstract

**Background:** DNA methylation is influenced by environmental factors and contributes to adverse modification of cancer risk and clinicopathological features.

**Methods:** A case-control study (402 newly diagnosed cases, 470 controls) was conducted to evaluate the effect of environmental factors and *OPRM1* methylation in peripheral blood leukocyte (PBL) DNA on the risk of breast cancer. A case-only study (373 cases) was designed to evaluate the effects of environmental factors on *OPRM1* methylation in tumor tissue and the relationship of methylation with clinicopathological features.

**Results:** We found a significant association between hypermethylation of *OPRM1* and the risk of breast cancer (OR = 1.914, 95%CI = 1.357–2.777). *OPRM1* hypermethylation in PBL DNA combined with low intake of vegetable, garlic, soybean, poultry, and milk; high pork intake; less regular sports and a high psychological stress index significantly increased the risk of breast cancer. Soybean intake (OR = 0.425, 95%CI: 0.231–0.781) and regular sports (OR = 0.624, 95%CI: 0.399–0.976) were associated with *OPRM1* hypermethylation in tumor DNA. *OPRM1* hypermethylation in tumor tissue was correlated with estrogen receptor (ER) (OR = 1.945, 95%CI: 1.262–2.996) and progesterone receptor (PR) (OR = 1.611, 95%CI: 1.069–2.427) negative status; in addition, *OPRM1* hypermethylation in PBL DNA was associated with human epidermal growth factor receptor 2 (HER-2) negative status (OR = 3.673, 95%CI: 1.411–9.564).

**Conclusion:** A healthy diet, psychosocial adaptability, and regular sports are very beneficial for breast cancer prevention and progress, especially for *OPRM1* hypermethylation carriers. Personalized treatment considering the correlation between *OPRM1* hypermethylation and ER and PR status may provide a novel benefit for breast cancer patients.

## Introduction

Breast cancer is one of the most common public health issues among women (1). According to an International Agency for Research on Cancer (IARC) report, ~2.1 million new female breast cancer cases and 626,800 cancer deaths occurred worldwide in 2018 (2). The Chinese cancer registry estimated 304,000 new breast cancer patients and 70,000 deaths among women in 2015 (2). Despite significant progress being made in treatment, breast cancer is still the main cancer challenge (3).

Breast cancer is a heterogeneous disease with distinct genetic, epigenetic and histopathological characteristics (4). DNA methylation as an important epigenetic process can regulate gene expression and influence the occurrence of cancers by transferring a methyl group to the C5 position of cytosine (5). Several studies have reported a link of peripheral blood leukocyte (PBL) DNA methylation with cancer diagnosis (6). Ji et al. (7) detected 5-methylcytosine (5-mdc) levels in PBL DNA in breast cancer cases and healthy controls and confirmed that leukocyte genome-wide DNA hypomethylation is independently associated with breast cancer risk.

DNA methylation can be altered by environmental factors, which in turn affect breast cancer risk (8). Tobacco exposure is associated with the methylation of several tumor suppressor genes in breast cancer tissues (9). Alcohol ingestion in animals is known to potentially inhibit folate-mediated methionine synthesis, disrupt key methylation processes and increase the risk of breast cancer (10). Moreover, DNA methylation is associated with specific clinical characteristics that affect the progress of tumors, such as estrogen receptor (ER), progesterone receptor (PR), and human epidermal growth factor receptor 2 (HER-2) (11). ER and PR are poorly expressed in normal breast cells, but ER alpha (ERα) expression increases at the very earliest stages of tumorigenesis, implying that dysregulation of ERα expression contributes to breast tumor formation (12). HER-2 is often overexpressed in a variety of epithelial-derived tumors and is closely related to the development and prognosis of breast cancer (13).

Opioid receptor mu 1 (*OPRM1)* gene encodes the mu opioid receptor (*MOR*), one of three opioid receptors in humans (14). *MOR* is the main target of opioid analgesics such as endogenous opioid peptides, endorphins, and enkephalins (15). Endogenous opioid systems have been implicated in a wide variety of physiological and pathophysiological actions (16). Partington et al. (17) reported that endogenous opioids are involved in neurocognitive processing of social pain and reward, and *OPRM1* SNP minor allele carriers are known to be sensitive to the negative effects of social stress (18). Increased methylation of *OPRM1* is associated with Alzheimer's disease, opioid exposure, alcohol and drug addiction, etc. (19–21). Zagon et al. (22) showed that endogenous opioids inhibit tumor growth, while opioid antagonists promote cancer. However, the relationship between *OPRM1* methylation and breast cancer risk has not been reported yet.

Therefore, this study was carried out to investigate the relationship of *OPRM1* methylation in PBL DNA, environmental factors, and their combinations and interactions with breast cancer risk. We also investigated the effects of environmental factors on *OPRM1* methylation in breast tumor DNA and the relationship between *OPRM1* methylation and clinicopathological features in tumor DNA and PBL DNA.

## Materials and Methods

### Study Subjects

A case-control study was designed. A total of 402 breast cancer patients newly diagnosed in the Third Affiliated Clinical Hospital of Harbin Medical University from 2010 to 2014 were enrolled; none of them had received radiation therapy or chemotherapy. In addition, 217 patients from the Department of Orthopedics and Ophthalmology of Harbin Medical University and 253 volunteers from Xiangfang District were assembled as the control group. The control group excluded subjects with a history of breast cancer; subjects with malignant or benign tumors; and pregnant, postpartum, and lactating women. Each participant donated 5 ml of fasting peripheral venous blood before surgery or at registration.

We also conducted a case-only study to analyze the effects of environmental factors on *OPRM1* methylation in tumor tissue DNA and the relationship between clinicopathological features and *OPRM1* methylation in both PBL and tumor DNA. Tissue specimens of 373 breast cancer patients were collected, frozen in liquid nitrogen as soon as possible, and immediately stored in a −80°C refrigerator.

### Ethics Approval and Consent to Participate

All procedures performed in studies involving human participants were granted from the ethical standards of the Human Research and Ethics Committee of Harbin Medical University and the 1964 Helsinki Declaration and its later amendments or comparable ethical standards. All subjects gave informed consent.

### Data Collection

All participants were interviewed face-to-face by trained investigators. The content of the questionnaire included demographic characteristics (age, marital status, education level, etc.), behavior (smoking, drinking, regular sports, diet, etc.), menstrual and reproductive history, etc. The clinical and pathological information of cancer patients was extracted from medical records, including the TNM stage, histological records, and other pathological results. We did our best to obtain a complete questionnaire from each participant, but there were still some subjects who did not answer individual questions. Therefore, the *n* value in the table is not completely corresponding to the sample size.

### Genomic DNA Extraction and Sodium Bisulfite Modification

DNA was extracted from blood samples and <25 mg of minced tumor tissues using the QIAamp DNA Blood Mini kit (Qiagen, Hilden, Germany) and PureLinkTM Genomic DNA Kit (Thermo Fisher Scientific, Carlsbad, USA), respectively. All steps were performed in accordance with the manufacturer's protocol, and purified DNA was stored at −80°C as soon as possible. Bisulfite conversion was performed according to the manufacturer's guidelines using 2 μg of DNA and the EpiTect Bisulfite Kit (Qiagen, Hilden, Germany). Before and after the transformation process, the amount of DNA was determined using a Nanodrop 2000 spectrophotometer (Thermo Fisher Scientific, Carlsbad, USA). The transformed DNA was also immediately stored at −80°C.

### Plasmid Construction and Standard Curve Dilution

According to the manufacturer's instructions, the TOPO TA Cloning for Sequencing kit (Invitrogen, USA) was used to transfer freshly purified, target-region-specific PCR products to TOP10 competent cells for cloning (23). The extracted plasmid DNA was confirmed by sequencing. Serial dilutions of 10–10^7^ copies of plasmid DNA were used to construct a standard curve for quantification.

### Quantification of DNA Methylation With Quantitative Methylation-Specific PCR

DNA methylation was measured by quantitative methylation-specific PCR technology (qMSP). Bisulfite-treated DNA from each specimen served as the template, and qMSP was performed with iTaq Universal SYBR Green Supermix (Bio Rad, America) and ABI 7500 real-time PCR amplifier (Applied Biosystems, Foster City, CA, USA) while strictly following the operating protocols.

Primers were designed for qMSP analysis using Primer Premier 5.0 software as follows: *OPRM1*: forward primer, 5′-CGGTTATTTATCGTTTGTAGGAGGAAACG-3′ reverse primer; 5′-ATCCAACAACGCTTCTATTCGAATCCG-3′. The internal reference was designed as follows: housekeeping gene (*MyOD*), forward primer, 5′-CCAACTCCAAATCCCCTCTCTAT-3′; reverse primer, 5′-TGATTAATTTAGATTGGGTTTAGAGAAGGA-3′.

For the amplification, 1 μl bisulfite converted DNA (5 ng) was added to 19 μl amplification Master Mix containing 10 μl SYBR Green Supermix, 0.6 μl of forward and reverse primer, and 7.8 μl RNAse-free water. The reaction protocol was as follows: (1) *OPRM1*, 6 min at 95°C, followed by 42 cycles of 95°C for 30 s, 58°C for 30 s, and 72°C for 30 s and (2) *MyOD*, 6 min at 95°C, followed by 42 cycles of 95°C for 30 s, 56°C for 30 s, and 72°C for 30 s. All the procedures were performed in duplicate, and 10–20% of the samples were redetected in our study.

The specific of amplification products were determined with melting curve analysis according to fluorescence data acquired during dissociation steps. Each sample's methylation level was calculated by the following formula: Methylation level = (quantity of target gene/quantity of housekeeping gene) × 100%.

### Statistical Analysis

Categorical and continuous variables were tested by a chi-square test and independent-sample *t*-test, respectively. Univariate and multivariate logistic regression analysis were used to calculate the crude and adjusted odds ratio (ORs) and 95% confidence interval (95%CIs). Combined and interaction effects were performed by crossover analysis (24) and multivariate logistic regression. Correlations between clinicopathological characteristics and the *OPRM1* methylation level in tumor DNA and PBL DNA were evaluated using ORs and 95%CIs derived from unconditional logistic regression. The effect of environment factors on *OPRM1* methylation in tumor tissue DNA was calculated using unconditional univariate and multivariate logistic regression. All statistical analyses were performed using SPSS version 24.0, with *P*-values of <0.05 considered statistically significant.

## Results

### Quality Evaluation of Quantitative Methylation-Specific PCR for *OPRM1*

**Figure 1:**
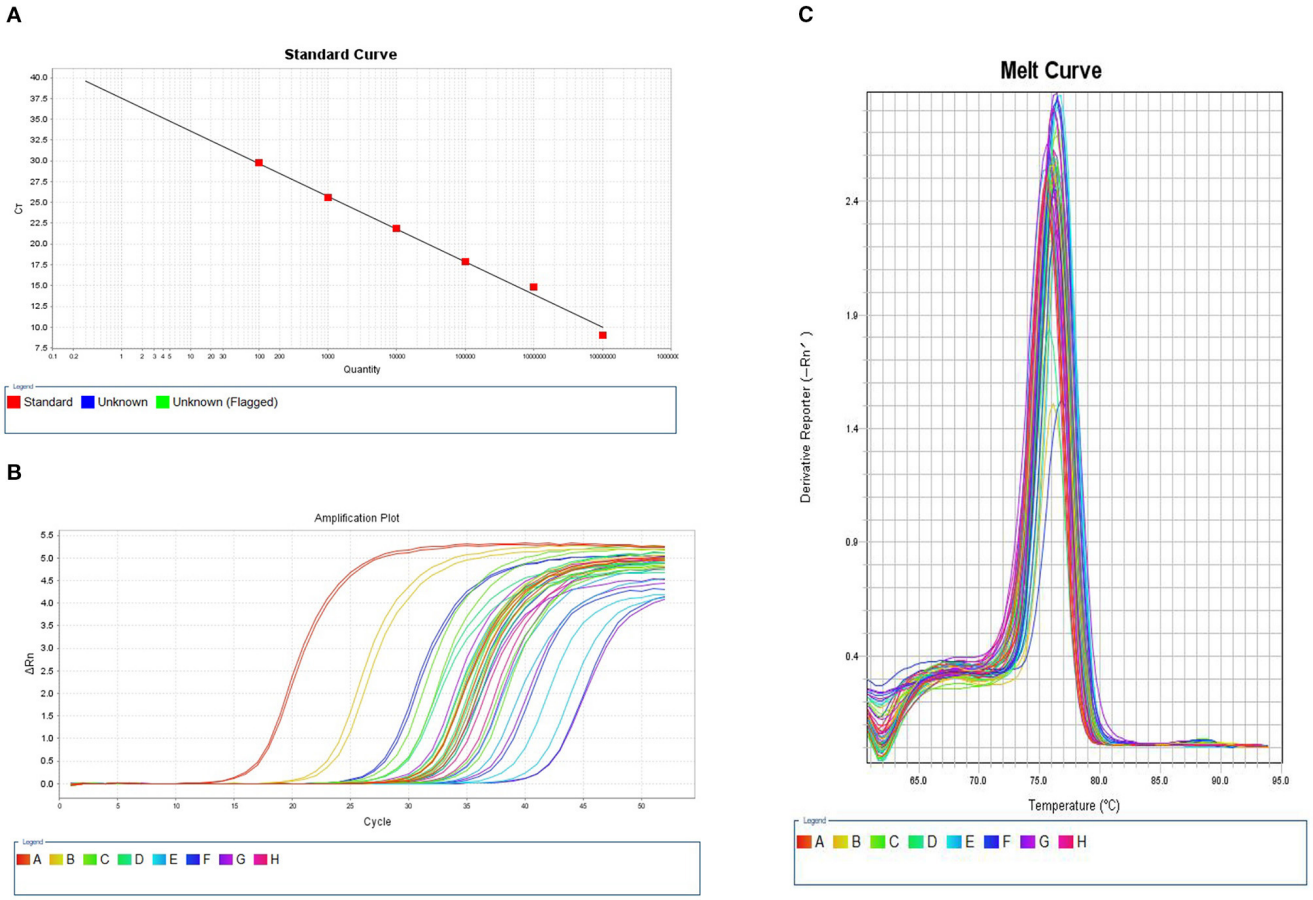
Quantitative methylation-specific PCR (qMSP) curves of *OPRM1* methylation. **(A)** Standard curve. The *R*^2^ value was 0.998, and the amplificationefficiency (EFF %) was 53.218%. **(B)** Amplification plot. **(C)** Melt curve. The melting peaks of serial dilutions of plasmid DNA and samples.

We constructed a standard curve using a 10–10^7^ copy number of plasmid DNA as template DNA in PCR process. Figure 1A shows the standard curve of quantitative methylation-specific PCR for *OPRM1*. The *R*^2^ value was 0.998, and the amplification efficiency (EFF %) was 53.218%. The signals of samples were compared to the standard curve. Figure 1B displays the amplification plot of serial dilutions of bisulfite modified, universal methylated DNA and samples. All samples were well-amplified, and the amplification plots reached a plateau. The melt curves of bisulfite modified universal methylated DNA and samples were also compared (Figure 1C).

### The Relationship Between *OPRM1* Methylation in PBL DNA and Breast Cancer Risk

A total of 402 PBL DNA samples from breast cancer patients and 470 control samples were analyzed. The distribution of basic demographic characteristics is shown in Supplementary Table 1. The differences were statistically significant in the categories of marital status (*P* = 0.040), educational level (*P* = 0.001), occupation (*P* = 0.000), and family history of cancer (*P* = 0.000). Therefore, these characteristics were adjusted as confounding factors in the following analyses. As shown in Table 1, by adjusting the marital status, educational level, occupation, and family history of cancer, we found a significant association between the hypermethylation of *OPRM1* in PBL DNA and the risk of breast cancer (OR = 1.914, 95%CI = 1.357–2.777, *P* = 0.000).

**Table 1:** Association of *OPRM1* methylation in peripheral blood leukocyte DNA and breast cancer risk.

**Methylation status^**a**^**	**Cases no. (%)**	**Controls no. (%)**	**OR_**crude**_**	**OR_**adj**_**
			**(95%CI)**	**(95%CI)**
Hypomethylation	79 (19.7)	160 (34.0)	1.000	1.000
Hypermethylation	322 (80.3)	310 (66.0)	2.104 (1.541–2.872)^b^	1.914 (1.357–2.777)^b^

*OR_crude_, odds ratio generated by univariate logistic regression; OR_adj_, odds ratio adjusted by marital status, education level, occupation, and family history of cancer*.

a *Methylation status, the methylation cut-off value (0.0076) was selected according to the maximum Yoden index*.

b *P < 0.0001*.

### Relationship of Environmental Exposure and Breast Cancer Risk

Univariate and multivariate logistic regression were used to analyze the relationship between environmental factors and breast cancer risk. As shown in Table 2, according to univariate logistic regression analysis, factors such as pork intake and psychological stress index are risk factors for breast cancer (*P* < 0.05). Intake of vegetable, garlic, poultry, milk, and soybean and regular sports are protective factors against breast cancer (*P* < 0.05). Subsequently, those environmental factors identified as significant were examined in the multivariate analyses. The results showed that vegetable intake (≥500 vs. <500 g/d, OR = 0.576, 95%CI: 0.407–0.816), garlic intake (≥1 vs. <1 time/week, OR = 0.468, 95%CI: 0.331–0.662), poultry intake (≥1 vs. <1 time/week, OR = 0.411, 95%CI: 0.281–0.601), milk intake (≥3 vs. <3 times/week, OR = 0.496, 95%CI: 0.335–0.732), regular sports (≥1 vs. <1 time/week OR = 0.604, 95%CI: 0.419–0.871), and soybean intake (≥1 vs. <1 time/week, OR = 0.500, CI: 0.291–0.859) were significantly associated with a decreased risk of breast cancer. A high intake of pork (≥1 vs. <1 time/week, OR = 2.956, 95%CI: 1.792–4.875) and a high psychological stress index (>15 vs. ≤15, OR = 1.938, 95%CI: 1.293–2.903) were significantly associated with an increased risk of breast cancer.

**Table 2:** Univariate and multivariate analysis for the association of environmental factors and the risk of breast cancer.

**Factors**	**Cases**	**Controls**	**Univariate analysis**		**Multivariate analysis**	
	**No. (%)**	**No. (%)**	**OR (95%CI)**	***P-*value**	**OR_**adj**_ (95%CI)**	***P-*value**
**Coarse grain (g/week)**						
<50	186 (56.2)	224 (48.1)	1.000			
≥50	145 (43.8)	242 (51.9)	1.090 (0.739–1.608)	0.663		
**Vegetable (g/day)**						
<500	235 (59.8)	209 (45.1)	1.000		1.000	
≥500	157 (40.2)	254 (54.9)	0.490 (0.335–0.715)	**0.000**	0.576 (0.407–0.816)	**0.002**
**Garlic (time/week)**						
<1	270 (67.5)	177 (37.9)	1.000		1.000	
≥1	131 (32.5)	290 (62.1)	0.444 (0.303–0.649)	**0.000**	0.468 (0.331–0.662)	**0.000**
**Pork (time/week)**						
<1	50 (12.5)	97 (20.7)	1.000		1.000	
≥1	351 (87.5)	372 (79.3)	3.374 (1.090–5.907)	**0.000**	2.956 (1.792–4.875)	**0.000**
**Poultry (time/week)**						
<1	221 (54.9)	196(41.9)	1.000		1.000	
≥1	181 (45.1)	272(58.1)	0.406 (0.270–0.610)	**0.000**	0.411 (0.281–0.601)	**0.000**
**Egg (No./week)**						
<3	169 (42.3)	212(45.1)	1.000			
≥3	232 (57.8)	258(54.9)	1.108 (0.748–1.641)	0.609		
**Milk (times/week)**						
<3	294 (73.9)	276 (59.1)	1.000		1.000	
≥3	104 (26.1)	191 (40.9)	0.378 (0.241–0.593)	**0.000**	0.496 (0.335–0.732)	**0.000**
**Sauerkraut (time/week)**						
<2	246 (61.3)	261 (55.9)	1.000			
≥2	155 (38.7)	207 (44.1)	0.797 (0.608–1.064)	0.102		
**Soybean (time/week)**						
<1	66 (16.8)	38 (8.1)	1.000		1.000	
≥1	327 (83.2)	429 (91.9)	0.436 (0.243–0.779)	**0.005**	0.500 (0.291–0.859)	**0.012**
**Coffee** ^**a**^						
No	374 (93.3)	434 (92.5)	1.000			
Yes	27 (6.8)	35 (7.5)	0.614 (0.207-1.822)	0.232		
**Carbonated drinks** ^**b**^						
No	383 (96.2)	440 (93.8)	1.000			
Yes	15 (3.8)	29 (6.2)	0.614 (0.207–1.822)	0.380		
**Juice** ^**b**^						
No	368 (92.4)	431 (91.9)	1.000			
Yes	30(7.6)	38(8.1)	0.830 (0.362–1.903)	0.660		
**Tea (time/week)**						
<1	337 (84.2)	397 (85.4)	1.000			
≥1	63 (15.8)	68 (14.6)	1.650 (0.945–2.881)	0.078		
**Smoking** ^**c**^						
No	347 (86.5)	415 (88.5)	1.000			
Yes	54 (13.5)	54 (11.5)	1.402 (0.855–2.298)	0.180		
**Oral contraceptive**						
No	344 (87.1)	420 (89.7)	1.000			
Yes	52 (12.9)	48 (10.3)	1.527 (0.507–4.583)	0.450		
**Alcohol (time/week)**						
<1	23 (6.3)	23 (5.3)	1.000			
≥1	344 (93.7)	411 (94.7)	1.239 (0.714–2.151)	0.446		
**Regular sports**^**e**^ **(time/week)**						
<1	257 (64.6)	253 (54.4)	1.000		1.000	
≥1	141 (35.4)	212 (45.6)	0.571 (0.375–0.870)	**0.009**	0.604 (0.419–0.871)	**0.007**
**Psychological stress score** ^**d**^						
≤15	69 (17.2)	149 (32.1)	1.000		1.000	
>15	332 (82.8)	315 (67.9)	1.635 (1.060–2.520)	**0.026**	1.938 (1.293–2.903)	**0.001**
**Menopause status**						
No	184 (46.8)	209 (44.5)	1.000			
Yes	208 (53.2)	261 (55.5)	0.877 (0.580–1.326)	0.534		

*OR_adj_, odds ratio generated by multivariate logistic regression*.

a *Coffee, Yes, drinking at least one cup per month for more than 3 months*.

b *Carbonated drinks, Juice, Yes, drinking at least one cup per week for more than 3 months*.

c *Smoking, Yes, smoking at least one per day for more than 6 weeks*.

d *Psychological stress score was evaluated using psychosocial stress survey for groups (PSSG)*.

e *Regular sports, regular physical exercise such as walking, running, fitness exercise, swimming, and jumping rope*.

*The bold values represent the results with statistical significance*.

### Combined and Interactive Effects of *OPRM1* Methylation and Environmental Factors in Breast Cancer

As shown in Table 3, comparing with the reference group, statistically significant combinations of *OPRM1* hypermethylation and low vegetable intake (<500 g/day, OR = 2.829, 95%CI: 1.751–4.572, *P* = 0.000), garlic intake (<1 time/week, OR = 5.762, 95%CI: 3.208–10.350, *P* = 0.000), soybean intake (<1 time/week, OR = 5.592, 95%CI: 2.905–10.764, *P* = 0.000), poultry intake (<1 time/week, OR = 4.173, 95%CI: 2.415–7.210, *P* = 0.002), milk intake (<3 times/week, OR = 5.166, 95%CI: 2.774–9.618, *P* = 0.000), and less sports (<1 time/week, OR = 4.114, 95%CI: 2.357–7.181, *P* = 0.000) were observed to be significantly related to increased breast cancer risk. In addition, we also found a significant combination of *OPRM1* hypermethylation and a high pork intake (≥1 time/week, OR = 3.628, 95%CI: 1.593–8.262, *P* = 0.000) and high psychological stress index (>15, OR = 5.340, 95%CI: 2.634–10.829, *P* = 0.000) for increased breast cancer risk. There was no significant interaction between *OPRM1* hypermethylation and any environmental factor in breast cancer.

**Table 3:** Combined and interactive effects between *OPRM1* methylation and environmental factors in breast cancer.

**Environment factors**	**Combined effects**			**Interactive effects**	
	**Hypomethylation**	**Hypermethylation**	***P*-value**	**OR_**i**_ (95%CI)**	***P-*value**
		**OR_**eg**_ (95%CI)**			
**Vegetable (g/day)**
≥500	1.000	1.706 (1.046–2.781)		1.000	
<500	1.303 (0.701–2.424)	**2.829 (1.751**–**4.572)**	**0.000**	0.786 (0.381–1.618)	0.513
**Garlic (time/week)**
≥1	1.000	2.066 (1.149–3.712)		1.000	
<1	2.601 (1.365–4.955)	**5.762 (3.208**–**10.350)**	**0.000**	0.932 (0.442–1.966)	0.854
**Soybean (time/week)**
≥1	1.000	1.857 (1.265–2.727)		1.000	
<1	1.811 (0.674–4.864)	**5.592 (2.905**–**10.764)**	**0.000**	0.601 (0.191–1.897)	0.386
**Pork (time/week)**				
<1	1.000	1.851 (0.743–4.612)		1.000	
≥1	1.834 (0.774–4.346)	**3.628 (1.593**–**8.262)**	**0.002**	1.069 (0.397–2.875)	0.895
**Poultry (g/week)**
≥1	1.000	2.825 (1.648–4.840)		1.000	
<1	2.901 (1.544–5.452)	**4.173 (2.415**–**7.210)**	**0.000**	1.964 (0.946–4.075)	0.070
**Milk (times/week)**
≥3	1.000	2.445 (1.271–4a.703)		1.000	
<3	3.409 (1.544–6.020)	**5.166 (2.774**–**9.618)**	**0.000**	1.443 (0.657–3.170)	0.361
**Regular sports**^**a**^ **(time/week)**
≥1	1.000	2.151 (1.211–3.822)		1.000	
<1	2.300 (1.218–4.344)	**4.114 (2.357**–**7.181)**	**0.000**	1.203 (0.576–2.514)	0.623
**Psychological stress score** ^**b**^
≤15	1.000	2.368 (1.099–5.103)		1.000	
>15	2.972 (1.398–6.321)	**5.340 (2.634**–**10.829)**	**0.000**	0.759 (0.319–1.806)	0.533

*OR_eg_: OR_genetic&environment_, combined effects of methylation and environmental factors*.

*OR_i_: OR_interaction_, interactive effects of methylation and environmental factors*.

a *Regular sports, regular physical exercise such as walking, running, fitness exercise, swimming, and jumping rope*.

b *Psychological stress score was evaluated using psychosocial stress survey for groups (PSSG)*.

*Bold values indicate significance after Bonferroni correction, the P-value after Bonferroni correction is 0.05/16 = 0.003125*.

### Effects of Exposure to Environmental Factors on *OPRM1* Methylation in Tumor Tissue DNA

A total of 373 patients with breast cancer were included in this analysis. The basic demographic characteristics for the hypermethylated and hypomethylated groups are shown in Supplementary Table 2. No significant difference in age, educational level, marital status, occupation, family history of cancer, or BMI distribution (all *P* > 0.05) between the *OPRM1* hypermethylated group and the hypomethylated group was observed.

Based on the results of univariate logistic regression, the history of estrogen therapy (yes vs. no, OR = 2.712, 95%CI: 1.163–6.325), soybean intake (≥1 vs. <1 time/week, OR = 0.420, 95%CI: 0.233–0.758), and regular sports (≥1 vs. <1 time/week, OR = 0.606, 95%CI: 0.392–0.938) are significantly related with *OPRM1* hypermethylation in tumor tissue DNA. After introducing these variables into a multivariate model, the soybean intake (≥1 vs. <1 time/week, OR = 0.425, 95%CI: 0.231–0.781) and regular sports (≥1 vs. <1 time/week, OR = 0.624, 95%CI: 0.399–0.976) were still significantly associated with *OPRM1* hypermethylation (Table 4).

**Table 4:** Relationship between environmental factors exposures and *OPRM1* methylation in breast cancer tissue DNA.

**Environmental factors**	***OPRM1***		**Univariate analysis**		**Multivariate analysis**	
	**Hypermethylation** **no. (%)**	**Hypomethylation** **no. (%)**	**OR (95%CI)**	***P-*value**	**OR (95%CI)**	***P-*value**
**Regular menstruation**						
Yes	141 (77.9)	136 (76.1)	1.000			
No	40 (2.1)	45 (24.9)	0.857 (0.527–1.395)	0.535		
**Menopause status**						
Yes	124 (68.5)	108 (60.0)	1.000			
No	57 (31.5)	72 (40.0)	0.690 (0.447–1.395)	0.092		
**History of estrogen therapy**						
No	8 (4.3)	20 (10.8)	1.000		1.000	
Yes	179 (95.7)	165 (89.2)	2.712 (1.163–6.325)	**0.021**	2.368 (0.916–6.118)	0.075
**History of breast disease** ^**a**^						
No	125 (68.7)	127 (71.3)	1.000			
Yes	57 (31.3)	51 (28.7)	1.136 (0.723-1.783)	0.581		
**Fine grain (g/day)**						
≤20	103 (56.0)	110 (60.4)	1.000			
>20	81 (44.0)	72 (39.6)	1.201 (0.793–1.821)	0.387		
**Coarse grain (g/week)**						
≤10	102 (55.7)	93 (51.1)	1.000			
>10	81 (44.3)	81 (48.9)	0.830 (0.550–1.253)	0.375		
**Fruit (g/week)**						
≤1,000	85 (45.7)	91 (50.0)	1.000			
>1,000	101 (54.3)	91 (50.0)	1.188 (0.789–1.789)	0.409		
**Pork (g/week)**						
<250	110 (61.5)	103 (56.9)	1.000			
≥250	69 (38.5)	78 (43.1)	0.828 (0.544–1.262)	0.380		
**Allium vegetable (times/week)**						
≤3	123 (66.5)	128 (70.7)	1.000			
>3	62 (33.5)	53 (29.3)	1.087 (0.720–1.642)	0.692		
**Fat meat (time/month)**						
≥1	116 (62.7)	120 (64.9)	1.000			
<1	69 (37.3)	65 (35.1)	0.911 (0.596–1.392)	0.665		
**Beef and mutton (time/month)**						
<1	99 (55.3)	109 (60.2)	1.000			
≥1	80 (44.7)	72 (39.8)	1.223 (0.805–1.860)	0.345		
**Fresh seafood (time/month)**						
<1	156 (87.6)	151 (83.4)	1.000			
≥1	22 (12.4)	30 (16.6)	0.846 (0.620–1.156)	0.295		
**Egg (times/week)**						
≤3	73 (39.7)	69 (37.5)	1.000			
>3	111 (60.3)	115 (62.5)	0.912 (0.600–1.388)	0.668		
**Milk (time/week)**						
<1	120 (65.9)	125 (70.6)	1.000			
≥1	62 (34.1)	52 (29.4)	1.242 (0.795–1.939)	0.341		
**Poultry (time/week)**						
<1	94 (52.2)	104 (57.1)	1.000			
≥1	86 (47.8)	78 (42.9)	1.220 (0.806–1.846)	0.347		
**Fried food (time/month)**						
<1	125 (67.2)	130 (70.9)	1.000			
≥1	61 (32.8)	54 (29.3)	1.175 (0.756–1.826)	0.474		
**Canned fruit (time/month)**						
<1	156 (83.4)	148 (80.4)	1.000			
≥1	31 (16.6)	36 (19.6)	0.817 (0.481–1.388)	0.455		
**Marine fish (time/month)**						
<1	114 (63.0)	114 (62.3)	1.000			
≥1	67 (37.0)	69 (37.7)	1.015 (0.865–1.190)	0.857		
**Vegetable (g/week)**						
≤500	109 (58.9)	123 (67.2)	1.000			
>500	76 (41.1)	60 (32.8)	1.429 (0.934–2.187)	0.100		
**Soybean (time/week)**						
<1	40 (21.5)	19 (10.3)	1.000		1.000	
≥1	146 (78.5)	165 (89.7)	0.420 (0.233–0.758)	**0.004**	0.425 (0.231–0.781)	**0.006**
**Sauerkraut (time/month)**						
<1	86 (46.2)	88 (47.8)	1.000			
≥1	100 (53.8)	96 (52.2)	1.066 (0.709–1.603)	0.759		
**Overnight food**^**b**^ **(times/week)**						
≤3	112 (60.9)	111 (60.7)	1.000			
>3	72 (39.1)	72 (39.3)	0.991 (0.652–1.507)	0.967		
**Smoking** ^**c**^						
No	160 (86.5)	162 (88.5)	1.000			
Yes	25 (13.5)	21 (11.5)	1.205 (0.648–2.241)	0.555		
**Drinking (time/week)**						
<1	143 (81.2)	145 (81.5)	1.000			
≥1	33 (18.8)	33 (18.5)	0.897 (0.673–1.195)	0.457		
**Regular sports**^**d**^ **(time/week)**						
<1	133(71.5)	108 (60.3)	1.000		1.000	
≥1	53 (28.5)	71 (39.7)	0.606 (0.392–0.938)	**0.025**	0.624 (0.399–0.976)	**0.039**
**Honey (time/week)**						
<1	109 (58.9)	113 (38.9)	1.000			
≥1	76 (41.1)	72 (61.1)	0.914 (0.603–1.385)	0.671		
**Tea (time/week)**						
≥1	18 (9.7)	22 (11.9)	1.000			
<1	167 (90.3)	163 (88.1)	1.252 (0.648–2.421)	0.504		

a *History of estrogen therapy, including lobular hyperplasia, fibroadenoma, and breast cysts*.

b *Overnight food, the vegetables, eggs, meat that have been cooked and left overnight*.

c *Smoking, Yes, smoking at least one per day for more than 6 weeks*.

d *Regular sports, regular physical exercise such as walking, running, fitness exercise, swimming, and jumping rope*.

*The bold values represent the results with statistical significance*.

### Relationship Between Clinicopathological Characteristics and *OPRM1* Methylation in PBL and Tumor Tissue DNA

The relationship between clinicopathological characteristics and *OPRM1* methylation in breast tumor tissue DNA and PBL DNA is shown in Table 5. We found that *OPRM1* hypermethylation in tumor tissue DNA was significantly correlated with ER status (negative vs. positive, OR = 1.945, 95%CI: 1.262–2.996) and PR status (negative vs. positive, OR = 1.611, 95%CI: 1.06–2.427). In addition, *OPRM1* hypermethylation in PBL DNA was significantly associated with HER-2 negative status (vs. positive, OR = 3.673, 95%CI: 1.411–9.564). No significant relation between *OPRM1* hypermethylation in either tumor tissue DNA or PBL DNA with the TNM stage, pathological type, tumor invasion status, lymphnode involved, metastasis status, histological type or P53 expression was found.

**Table 5:** Correlation between clinicopathological characteristics and *OPRM1* methylation in breast tumor tissue DNA and peripheral blood leukocyte DNA.

**Character of clinical pathology**	**Tumor tissue** ***DNA***				**Peripheral blood leukocyte DNA**.			
	**Hypermethylated no. (%)**	**Hypomethylated no. (%)**	**OR_**crude**_ (95%CI)^**a**^**	***P-*value**	**Hypermethylated no. (%)**	**Hypomethylated no. (%)**	**OR_**crude**_ (95%CI)^**a**^**	***P-*value**
**TNM stages**						
I	44 (23.5)	45 (24.2)	1.000		24 (35.8)	90 (33.3)	1.000	
II	96 (51.3)	98 (52.7)	1.002 (0.607–1.655)	0.994	37 (55.2)	153 (56.7)	1.103 (0.620–1.961)	0.739
III–IV	47 (25.1)	43 (23.1)	1.118 (0.622–2.009)	0.701	6 (9.0)	27 (10.0)	1.200 (0.445–3.238)	0.719
**Tumor invasion**						
T1	61 (32.6)	78 (42.2)	1.000		126 (46.7)	31 (46.3)	1.000	
T2–T4	126 (67.4)	107 (57.8)	1.506 (0.987–2.298)	0.058	144 (53.3)	36 (53.7)	0.984 (0.576–1.683)	0.953
**Lymphnodes involved**						
N0	98 (52.4)	102 (54.8)	1.000		134 (49.8)	30 (44.8)	1.000	
N1/N2	89 (47.6)	84 (45.2)	1.103 (0.734–1.657)	0.638	135 (50.2)	37 (55.2)	0.817 (0.477–1.398)	0.461
**Metastasis status**						
M0	177 (94.7)	180 (96.8)	1.000		258 (95.6)	63 (96.9)	1.000	
M1	10 (5.3)	6 (3.2)	1.695 (0.603–4.763)	0.317	12(4.4)	2(3.1)	1.465 (0.320–6.713)	0.623
**Histological type**						
Non-invasive	48 (25.8)	48 (28.3)	1.000		18 (6.9)	3 (4.5)	1.000	
Invasive	138 (47.2)	138 (71.7)	1.000 (0.628–1.591)	1.000	243 (93.1)	63 (95.5)	1.556 (0.444–5.447)	0.490
**ER status**						
Positive	134 (72.0)	106 (57.0)	1.000		15 (7.1)	4 (7.8)	1.000	
Negative	52 (28.0)	80 (43.0)	1.945 (1.262–2.996)	**0.003**	196 (92.9)	47 (92.2)	1.112 (0.353–3.505)	0.856
**PR status**						
Positive	109 (58.6)	87 (46.8)	1.000		19 (9.9)	6 (12.2)	1.000	
Negative	77 (41.4)	99 (53.2)	1.611 (1.069–2.427)	**0.023**	173 (90.1)	43 (87.8)	1.271 (0.478–3.3.74)	0.613
**HER2 expression**						
Positive	124 (66.7)	124 (67.0)	1.000		13 (6.4)	8 (20.0)	1.000	
Negative	62 (33.3)	61 (33.0)	1.061 (0.660–1.566)	0.941	191 (93.6)	32 (80.0)	3.673 (1.411–9.564)	**0.008**
**Molecular subtype** ^**b**^						
Luminal A	24 (12.9)	25 (13.5)	1.000		2 (1.4)	1 (3.1)	1.000	
Luminal B	38 (20.4)	36 (19.5)	1.100 (0.534–2.264)	0.797	11 (7.9)	7 (21.9)	0.786 (0.059–10.377)	0.855
HER-2 enriched	98 (52.7)	72 (38.9)	0.418 (0.750–2.682)	0.283	114 (81.4)	21 (65.6)	2.714 (0.235–31.302)	0.423
Basal-like	26 (14.0)	52 (28.1)	0.521 (0.251–1.083)	0.810	13 (9.3)	3 (9.4)	2.167 (0.144–35.528)	0.576
**P53**						
Negative	127 (69.8)	112 (71.7)	1.000		34 (33.0)	12 (36.4)	1.000	
Positive	55 (30.2)	52 (28.3)	1.099 (0.701–1.725)	0.680	69 (67.0)	21 (63.6)	1.160 (0.511–3.632)	0.723

a *OR_crude_, odds ratio generated by univariate logistic regression; 95%CI, 95% confidence interval*.

b *Subtypes were classified by immunohistochemically surrogates as basal-like (ER-, PR-, HER-2–, triple-negative), luminal A (ER and/or PR+, HER-2–), luminal B (ER and/or PR+, HER-2+), or HER-2 enriched (ER and PR–, HER-2+)*.

*The bold values represent the results with statistical significance*.

## Discussion

Accumulating research has shown the importance of DNA methylation in the impact of environmental factors on breast cancer risk, and this epigenetic mechanism is also associated with specific clinicopathological features (25). In this study, we discovered for the first time a series of valuable associations between *OPRM1* methylation and breast cancer. First, we found that *OPRM1* hypermethylation in PBL DNA increased the breast cancer risk. Furthermore, *OPRM1* hypermethylation combined with low intake of vegetable, garlic, soybean, poultry, and milk; less regular sports; and a high pork intake and high psychological stress index significantly increased the risk of breast cancer. We also found that soybean intake and regular sports reduced *OPRM1* methylation in tumor tissue. Additionally, *OPRM1* hypermethylation in tumor tissue DNA was significantly correlated with ER and PR negative status, and hypermethylation in PBL DNA was significantly associated with HER-2 negative status.

Some studies have indicated an association between psychological stress and breast cancer risk, which is consistent with our results (26). Our study found that a high psychological stress index (>15) increased the risk of breast cancer (OR = 1.938, 95%CI: 1.293–2.903). Stress hormone signaling may induce DNA damage and promote tumorigenesis by producing reactive oxygen species (ROS)/reactive nitrogen species (RNS) and interfering with DNA repair processes (27). Additionally, we found that high psychological stress combined with *OPRM1* hypermethylation in PBL DNA increased the breast cancer risk with a strong correlation (OR = 5.340, 95%CI: 2.634–10.829). Guerrero-Alba et al. (28) reported that psychological stress factors can significantly reduce endogenous opioid levels, block the binding of endogenous opioids to receptors, and thus lead to harmful events. Psychological stress may be correlated with hypermethylation of *OPRM1* and low expression of *OPRM1*-encoded mu-opioid receptors, which promote the occurrence of breast cancer. Thus, we suggest that individuals with *OPRM1* hypermethylation in PBL DNA maintain a good psychological state to prevent breast cancer.

Steensberg et al. (29) reported that increased exercise contributes to the upregulation of IL-6, which enhances the levels of anti-inflammatory cytokines IL-1α and IL-10. In addition, IL-6 inhibits the expression of tumor necrosis factor-α. According to our results, regular sports can reduce the risk of breast cancer significantly (OR = 0.604, 95%CI: 0.419–0.871). In particular, comparing with the individuals of *OPRM1* hypomethylation in PBL DNA and regular sports, we found that less frequent sports (<1 time/week) combined with *OPRM1* hypermethylation in PBL DNA increased breast cancer risk with a strong association (OR = 4.114, 95%CI: 2.357–7.181). Arida et al. (30) randomly divided rats into acute (7 days) and chronic (30 days) exercise groups and a control group and observed higher opioid receptor binding in the acute-exercise animals; they concluded that physical exercise can stimulate the release of endogenous opioid substances. By inference, less sports and hypermethylation of *OPRM1* may have combined action on the inhibition of endogenous opioid substances and increased the risk of breast cancer. Thus, we encourage people with *OPRM1* hypermethylation in PBL DNA to increase their physical activity to stay healthy. Strikingly, we also found that regular sport is associated with a decreased *OPRM1* methylation level in breast cancer tissues (OR = 0.624, 95%CI: 0.399–0.976). All of these results suggest that regular sports can not only reduce the risk of breast cancer directly but also indirectly change the individual's susceptibility to breast cancer through modification of DNA methylation.

In addition, many dietary factors are also related to breast cancer risk (31). We found that vegetable, garlic, poultry, and milk intake are protection factors and that pork intake is a risk factor for breast cancer (*P* < 0.05). Vegetables are rich in dietary fiber, which may decrease reabsorption in the gut of estrogen excreted in the biliary system (32). Garlic contains organosulfur compounds, of which diallyl trisulfide inhibits ER-α activity in human breast cancer cells (33). Milk is rich in vitamin D; Xie et al. (34) found that vitamin D analogs suppress IGF-I signaling and promote apoptosis in breast cancer cells. The protective effect of poultry can be explained by its amino acid content; for example, n-3 fatty acids inhibit tumor growth and metastasis by the effect of impaired angiogenesis (35). However, pork intake may increase breast cancer risk due to dietary heme iron, fat and N-glycolylneuraminic acid, which are indicated to possibly increase tumor formation (36). A combination of low vegetable, garlic, poultry, and milk intake and high pork intake and *OPRM1* hypermethylation in PBL DNA was observed in our study to be significantly associated with an increased risk of breast cancer (*P* < 0.05). Therefore, we also encourage that women with *OPRM1* hypermethylation should increase their intake of vegetable, garlic, milk and poultry and reduce their intake of pork to prevent breast cancer.

Genistein has been identified as the predominant isoflavone in soybean, which can prevent abundant cell proliferation or abnormal angiogenesis by inhibiting RTK-mediated signaling pathways (37). Zhu et al. (38) found higher consumption of soy protein decreased breast cancer risk significantly (OR = 0.46, 95%CI = 0.24–0.88). And the Shanghai Breast Cancer Survival Study, a large, population-based cohort study of 5,042 female breast cancer survivors in China, found that soy food consumption was significantly associated with decreased risk of death (HR = 0.71, 95%CI, 0.54–0.92) and recurrence (HR = 0.68,95%CI, 0.54–0.87) (39). We found that soybean intake reduced the risk of breast cancer (OR = 0.500, 95%CI: 0.291–0.859) and that low soybean intake combined with *OPRM1* hypermethylation increased the risk of breast cancer (OR = 5.592, 95%CI: 2.905–10.764). Moreover, soybean intake reduced *OPRM1* methylation in tumor tissue (OR = 0.425, 95%CI: 0.231–0.781). However, Katrin Sak reviewed the existing literature and reported that intake of isoflavones can be associated with a decrease in breast tumorigenesis only in Asian countries where the consumption of soy foods is high but not among Western women with significantly lower ingestion amounts (40). Some other studies revealed that the incidence of hormone-dependent breast cancer is much lower in the Asian population than in the United States and Europe (41). Soybean consumption may be responsible in part for lower levels of hormones and decreased rates of breast cancer in women in Asia compared with Western populations (42). Therefore, as for the inconsistent findings regarding soybeans and breast cancer risk, larger epidemiological studies involving multiracial populations are still needed to confirm the effect of genes and soy on breast cancer risk.

A clinicopathology staging system including ER, PR, and HER-2 was previously validated for the treatment and prognostic value of breast cancer in patients with breast cancer (43). Zagon et al. (44), Gach et al. (45), and Hatzoglou et al. (46) reported that the expression of *OPRM1* is related to ER positivity, which leads to ERα activation through MOR activation and mediates its translocation to plasma membrane, synergistically inducing the proliferation of breast cancer cells. *OPRM1* hypermethylation leads to a decrease in its gene expression, which also weakens ER activity. Similarly, in our study, we found that *OPRM1* hypermethylation was significantly associated with ER negative status and PR negative status in tumor tissue DNA (*P* < 0.05). As Dunnwald et al. (47) reported, ER- and PR-positive status in tumors not only improves the therapeutic effect but also increases the survival rate, so directional therapy plays a vital role in adjuvant breast cancer treatment. Although the mechanism of the association between *OPRM1* hypermethylation and ER/PR-negative status remains to be investigated, as does the association between *OPRM1* hypermethylation in PBL DNA and HER-2 negative status, our results may provide new ideas for the treatment of breast cancer patients through further studies on epigenetic regulation. Targeted induction of *OPRM1* methylation to regulate gene expression and design of possible therapeutic drugs may provide a novel remedy for breast cancer patients.

There are still some limitations with the interpretation of the broad results in our research. First, recall bias when collecting environmental factor data is difficult to avoid. In addition, all participants answered the same questionnaire, but different questions were omitted by different participants, so that the *n* value in the table could not correspond to the sample size. Second, the information about dietary factors in the questionnaire was divided into only two different frequencies, and the analysis of exposure dose was not detailed enough to give quantitative results. The sample size adopted in our subgroup analysis was relatively small, and the results need to be verified by a study with a larger sample size. The relationship between *OPRM1* methylation and increased breast cancer risk is based on the results of population epidemiological studies. The mechanism of *OPRM1* methylation and tumorigenesis based on *in vitro* studies still needs to be further developed and verified. It is also necessary to carry out *in vivo* interventional experiment in *OPRM1* hypermethylation population to verify the effect of individualized prevention.

## Conclusion

This study identified that *OPRM1* hypermethylation in PBL DNA is correlated with increased risk of breast cancer. A healthy diet with sufficient intake of vegetable, garlic, soybean, poultry and milk; a low pork intake; regular sports and healthy psychosocial adaptability is very beneficial for breast cancer prevention, especially for *OPRM1* hypermethylation carriers. Personalized promotion on regular sports and soybean intake and precision treatment based on DNA methylation markers (*OPRM1*) should be encouraged with the consideration of the correlation between *OPRM1* hypermethylation and ER/PR, HER-2 negative status in breast cancer.

## Data Availability Statement

The original contributions presented in the study are included in the article/Supplementary Material, further inquiries can be directed to the corresponding authors.

## Ethics Statement

The studies involving human participants were reviewed and approved by Human Research and Ethics Committee of Harbin Medical University. The patients/participants provided their written informed consent to participate in this study.

## Author Contributions

FW contributed to the study design. LL and SL contributed to methylation detection, data analysis, and drafting the manuscript. NZ contributed to data analysis. SQ, JD, YC, and CW contributed to data collection. YG, ZH, and YY contributed to data interpretation. YZ contributed to manuscript revision. All authors contributed to the article and approved the submitted version.

## Funding

This work was supported by grants from National Nature Science Foundation of China (Nos. 81202262 and 81773503), Postdoctoral Science Special Foundation of China (2013T60390), and Dr. Wu Lien-teh Science Foundation of Harbin Medical University (WLD-QN1106).

## Conflict of Interest

The authors declare that the research was conducted in the absence of any commercial or financial relationships that could be construed as a potential conflict of interest.

## Publisher's Note

All claims expressed in this article are solely those of the authors and do not necessarily represent those of their affiliated organizations, or those of the publisher, the editors and the reviewers. Any product that may be evaluated in this article, or claim that may be made by its manufacturer, is not guaranteed or endorsed by the publisher.
