# Supplementary material for: Diet, Sports, and Psychological Stress as Modulators of Breast Cancer Risk: Focus on OPRM1 Methylation
Source: Front Nutr. 2021 Dec 8;8:747964. doi: 10.3389/fnut.2021.747964 (PMC8744450; doi:10.3389/fnut.2021.747964)
Supplement: Supplementary file 1 [file Table_1.DOCX]

| **Supplemental Table 1. Demographic characteristics of breast cancer patients and controls in peripheral blood leukocyte DNA** | | | |
| --- | --- | --- | --- |
| **Characteristics^a^** | **Cases**  **No. (%)** | **Controls**  **No. (%)** | ***P-*value** |
|  | 402 | 470 |  |
| **Age (year)** | | | 0.356 |
| <40 | 42 (10.7) | 54 (11.5) |  |
| 40-49 | 140 (34.8) | 152 (32.3) |  |
| 50-59 | 140 (34.8) | 150 (31.9) |  |
| ≥60 | 79 (19.7) | 114 (24.3) |  |
| Means±SD | 50.91±9.98 | 51.93±10.05 |  |
| **Marital status^b^** | | | **0.040** |
| Single | 42 (10.5) | 31 (6.6) |  |
| Married | 358 (89.5) | 437 (93.4) |  |
| **Educational level** | | | **0.001** |
| Primary and below | 92 (22.9) | 136 (29.1) |  |
| Junior high school | 144 (35.8) | 154 (32.9) |  |
| Senior middle school | 97 (24.4) | 136 (29.1) |  |
| College and above | 68 (16.9) | 42 (9.0) |  |
| **Occupation** | | | **0.000** |
| Mental worker | 99 (27.1) | 96 (24.3) |  |
| Manual worker | 182 (49.9) | 262 (66.3) |  |
| Mixed | 84 (23.0) | 37 (9.4) |  |
| **Family history of cancer** | | | **0.000** |
| Yes | 112 (28.3) | 66 (15.9) |  |
| No | 284 (71.7) | 348 (84.1) |  |
| **BMI^c^ (kg/m^2^)** | | | 0.155 |
| <18.5 | 11 (2.7) | 19 (4.1) |  |
| 18.5-23.0 | 174 (43.4) | 176 (37.6) |  |
| ≥23.0 | 215 (53.9) | 273 (58.3) |  |
| Means±SD | 24.15±3.83 | 23.88±4.91 |  |

^a^Missing Data: Age: 1 case, 2 controls; Marriage status: 10 cases, 13 controls; Cultural level: 2 cases, 13 controls; BMI: 6 cases, 20 controls; Family history: 58 cases, 39 controls.

^b^Marrital status: Single is the person who has not married. Married is the person who has married or cohabitated, including widowed (not remarried), separated (due to discord or long-distance), and divorced (not remarried).

^c^BMI: body mass index (weight/height^2^).
