# Supplementary material for: Diet, Sports, and Psychological Stress as Modulators of Breast Cancer Risk: Focus on OPRM1 Methylation
Source: Front Nutr. 2021 Dec 8;8:747964. doi: 10.3389/fnut.2021.747964 (PMC8744450; doi:10.3389/fnut.2021.747964)
Supplement: Supplementary file 2 [file Table_2.DOCX]

**Supplemental Table 2. Demographic characteristics of breast cancer patients with tumor tissue DNA**

| **Demographic characteristics^a^** | ***OPRM1* methylation^b^** | | ***P-*value** |
| --- | --- | --- | --- |
|  | **Hypermethylation**  **No. (%)** | **Hypomethylation No. (%)** |  |
|  | 187 | 186 |  |
| **Age (year)** | | | 0.359 |
| <50 | 81 (43.5) | 78 (42.6) |  |
| 50-59 | 60 (32.3) | 70 (38.3) |  |
| ≥60 | 45 (24.2) | 35 (19.1) |  |
| Means±SD | 52.38±9.2 | 52.86±9.01 |  |
| **Marital status^c^** | | | 0.847 |
| Single | 15 (8.9) | 14 (7.6) |  |
| Married | 169 (91.8) | 170 (92.4) |  |
| **Educational level** | | | 0.674 |
| Elementary school and below | 50 (27.2) | 56 (30.1) |  |
| Middle/high School | 117 (63.6) | 110 (59.1) |  |
| University and above | 17 (9.2) | 20 (10.8) |  |
| **Family history of cancer** | | | 0.878 |
| Yes | 40 (21.5) | 39 (21.0) |  |
| No | 145 (78.5) | 147 (79.0) |  |
| **Occupation** | | | 0.309 |
| Mental worker | 35 (18.7) | 25 (13.6) |  |
| Manual workers | 72 (38.5) | 82 (44.6) |  |
| Mix | 80 (42.8) | 77 (41.8) |  |
| **BMI^d^ (kg/m^2^)** | | | 0.466 |
| <18.5 | 4 (2.2) | 8 (4.3) |  |
| 18.5-22.9 | 69 (37.7) | 64 (35.0) |  |
| ≥23 | 110 (60.1) | 111 (60.7) |  |
| Means±SD | 23.82±3.31 | 24.34±4.09 |  |

^a^Missing value: Age, 4. Marital status, 5. Education level, 3. Family history, 2. Occupation, 2. BMI, 7.

^b^*OPRM1* methylation status, the methylation cut-off value was 20.8%, selected according to the 50% percentile.

^c^Marrital status: Single is the person who has not married. Married is the person who has married or cohabitated, including widowed (not remarried), separated (due to discord or long-distance), and divorced (not remarried).

^d^BMI: body mass index (weight/height^2^).
